# *Gain-of-function* defects of astrocytic Kir4.1 channels in children with autism spectrum disorders and epilepsy

**DOI:** 10.1038/srep34325

**Published:** 2016-09-28

**Authors:** Federico Sicca, Elena Ambrosini, Maria Marchese, Luigi Sforna, Ilenio Servettini, Giulia Valvo, Maria Stefania Brignone, Angela Lanciotti, Francesca Moro, Alessandro Grottesi, Luigi Catacuzzeno, Sara Baldini, Sonia Hasan, Maria Cristina D’Adamo, Fabio Franciolini, Paola Molinari, Filippo M. Santorelli, Mauro Pessia

**Affiliations:** 1 Clinical Neurophysiology Laboratory, Department of Developmental Neuroscience, IRCCS Fondazione Stella Maris, Via dei Giacinti 2, 56128–Pisa, Italy; 2 Molecular Medicine Laboratory, Department of Developmental Neuroscience, IRCCS Fondazione Stella Maris, Via dei Giacinti 2, 56128–Pisa, Italy; 3 Department of Cell Biology and Neuroscience, Istituto Superiore di Sanità, Viale Regina Elena 299, 00161–Rome, Italy; 4 School of Medicine, Section of Physiology & Biochemistry, Department of Experimental Medicine, Piazzale Gambuli, University of Perugia, 06132–Perugia, Italy; 5 SuperComputing Applications and Innovation–CINECA, Via dei Tizii, 6, 00185–Roma, Italy; 6 Department of Chemistry, Biology and Biotechnology, University of Perugia, Piazzale Gambuli, 06132–Perugia, Italy; 7 Faculty of Medicine, Department of Physiology & Biochemistry, University of Malta, MSD 2080–Msida, Malta; 8 Department of Pharmacology, Istituto Superiore di Sanità, Viale Regina Elena 299, 00161–Rome, Italy

## Abstract

Dysfunction of the inwardly-rectifying potassium channels Kir4.1 (*KCNJ10*) represents a pathogenic mechanism contributing to Autism-Epilepsy comorbidity. To define the role of Kir4.1 variants in the disorder, we sequenced *KCNJ10* in a sample of affected individuals, and performed genotype-phenotype correlations. The effects of mutations on channel activity, protein trafficking, and astrocyte function were investigated in *Xenopus laevis* oocytes, and in human astrocytoma cell lines. An *in vivo* model of the disorder was also explored through generation of *kcnj10a* morphant zebrafish overexpressing the mutated human *KCNJ10*. We detected germline heterozygous *KCNJ10* variants in 19/175 affected children. Epileptic spasms with dysregulated sensory processing represented the main disease phenotype. When investigated on astrocyte-like cells, the p.R18Q mutation exerted a *gain-of-function* effect by enhancing Kir4.1 membrane expression and current density. Similarly, the p.R348H variant led to gain of channel function through hindrance of pH-dependent current inhibition. The frequent polymorphism p.R271C seemed, instead, to have no obvious functional effects. Our results confirm that variants in *KCNJ10* deserve attention in autism-epilepsy, and provide insight into the molecular mechanisms of autism and seizures. Similar to neurons, astrocyte dysfunction may result in abnormal synaptic transmission and electrical discharge, and should be regarded as a possible pharmacological target in autism-epilepsy.

Dysfunction of the astrocyte K^+^ channel Kir4.1, encoded by the *KCNJ10* gene (OMIM602208), is a common substrate to a number of neuronal phenotypes in several syndromic or non-syndromic neurodevelopmental disorders presenting with broad clinical manifestations and encompassing movement disorders, intellectual disability, autism spectrum disorders (ASD), and seizures^1^. Previous findings from clinical and functional investigations of *KCNJ10* mutations (p.R18Q and p.V84M) in two unrelated families^2^ indicated that seizures, intellectual disability, and ASD (namely, the autism-epilepsy phenotype, AEP) were the main clinical presentation of patients with *gain-of-function* (GoF) mutations of the Kir4.1 channel. Heterologous expression of the variants in *X. laevis* oocytes showed that an increase in single-channel conductance was clearly the cause of the GoF in p.V84M mutant channels. In the same cell model, we observed an increase in homomeric and heteromeric current amplitudes with no changes in single-channel properties for mutant p.R18Q^2^, suggesting an enhanced surface expression as a possible, yet unexplored, GoF mechanism.

Following up on these preliminary findings, we have screened a larger sample of ASD individuals, either with or without comorbid epilepsy/EEG abnormalities, in order to establish the relative frequency of Kir4.1 variants in the clinical condition, and to draw further genotype-phenotype correlations. To explore in more depth the consequences of new and already reported Kir4.1 variants, we have also assessed *in vitro* the effects of mutations on the complex functioning of astrocyte-neuron machinery, and their impact on clinical phenotypes *in vivo*.

## Results

### Clinical and genetic findings

The molecular screening of *KCNJ10* in 175 affected children revealed three missense heterozygous variants (namely, p.R18Q, p.V84M, and p.R348H) in a total of 9/175 (5.1%) individuals. The three *KCNJ10* variants were not found in any of our control DNA samples. Table 1 summarizes the main clinical findings of the patients harbouring the above mutations in *KCNJ10*. A more detailed clinical description is also reported in the Supplementary Data.

The c.53G > A (p.R18Q) variant in the cytoplasmic N-terminus domain was detected in 7/175 (4%) patients, five of whom were in the AEP subgroup. Seizures, when present, were well controlled under anti-epileptic drugs in all patients carrying the mutation p.R18Q. They displayed in addition a clear disorder of sensory processing regulation (hypersensitive, hyposensitive, sensory stimulation-seeking types). Among carrier relatives, two had clinical neuropsychiatric features. These included obsessive-compulsive symptoms and mood disorder in the mother of our original twin set^2^, and focal EEG abnormalities as well as subclinical manifestations of autism involving social motivation, expressiveness, and flexibility/range of interest areas, assessed by the BPASS (Broader Phenotype Autism Symptom Scale)^3^, in another parent.

The novel c.1043G > A (p.R348H) (*rs146396982*), located at the cytoplasmic C-terminus domain of the Kir4.1 protein, was detected in one girl with a history of epileptic spasms, beginning at four months of age, and multifocal EEG abnormalities. Spasms remitted without treatment two months later in connection with a febrile illness, and were followed by the development of a severe intellectual disability and ASD. The p.R348H variant was inherited from her father, who displayed severe anxiety disorder with panic attacks, and focal abnormalities on EEG recordings.

Lastly, we detected the c.811C > T (p.R271C) (*rs1130183*) in 10/175 (5.7%) individuals. This change is trusted as a neutral polymorphism because of its high frequency (MAF 0.04567) in polymorphic exome database (ExAC, exac.broadinstitute.org/) and in the 1000G dataset (www.1000genomes.org), and a benign prediction *in silico* using PolyPhen-2 (genetics.bwh.harvard.edu/pph2/), SIFT (SIFT, http://sift.jcvi.org/), and PANTHER (www.pantherdb.org/tools/csnpScoreForm.jsp). Moreover, in our in house controls the p.R271C had a MAF of 0.0521.

### *KCNJ10* variants vs wild-type

To explore correlations between variants (namely, p.R18Q, p.V84M, and p.R348H) in *KCNJ10* and phenotypic features, we compared wild-type (WT) individuals [166/175 (94.9%)] with those harbouring rare variants in *KCNJ10* [9/175 (5.1%)] (Table 2). The two groups were comparable with respect to age, gender, ASD diagnosis, language and cognitive development, frustration intolerance, self/hetero injurious behaviours, sleep disorders, presence of macrocephaly, and family history for epilepsy and/or ASD (Supplementary Table S1). In addition, they did not differ for the rate of seizure history, nor in the occurrence, type and site of EEG abnormalities. We found that *KCNJ10* variants were significantly associated with epileptic spasms (χ^2^ = 11.131, df = 2, p = 0.004), with a particularly good prognosis of the epilepsy outcome. Finally, individuals with *KCNJ10* variants showed a lower frequency of stereotyped behaviours (χ^2^ = 4.217, df = 1, p = 0.040), and a higher occurrence of regulation disorders of sensory processing (χ^2^ = 4.346, df = 1, p = 0.037) (see Table 2 for details). Similar results were also seen when children harbouring the neutral p.R271C were removed from the WT group (see Supplementary Tables S2 and S3).

### Functional findings

#### The p.R18Q mutation enhances membrane expression of Kir4.1 channels in astrocyte-like cells

**Figure 1:**
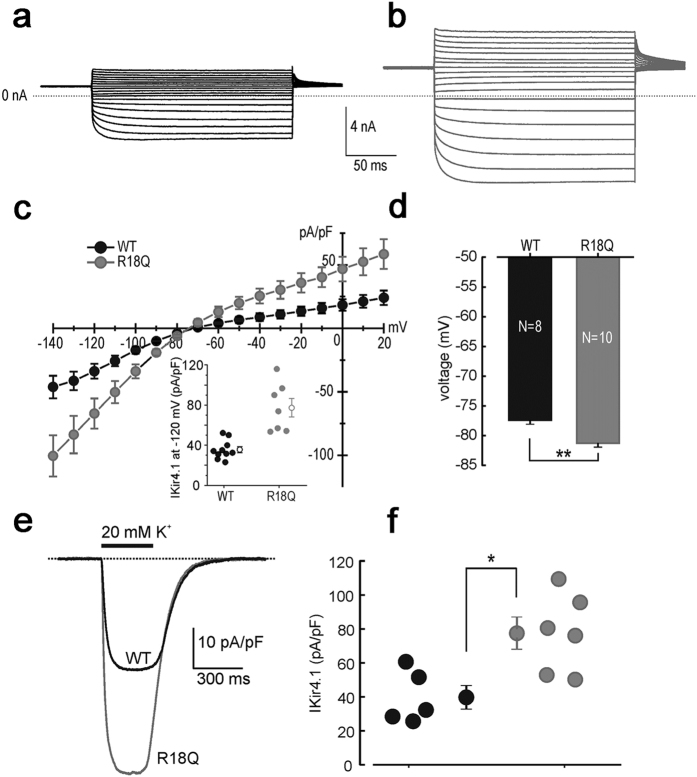
Whole-cell Kir4.1 currents recorded from cells expressing WT and R18Q channels. Sample current traces for (**a**) WT and (**b**) R18Q channels expressed in U251 astrocytoma cells, obtained in response to voltage steps from −140 to +20 mV in 10 mV steps, from a holding potential of −30 mV. (**c**) Mean current density (pA/pF) as a function of voltage obtained from cells expressing the indicated channels. Inset: scatter plot of the single-cell current densities at −120 mV. The open symbols represent the mean ± SEM. (**d**) The resting membrane potentials were evaluated in current clamp conditions from cells expressing WT (black bar) or R18Q (grey bar) channels (data are mean ± SEM; **p < 0.01). (**e**) Sample inward-current densities evoked by puffs of an external solution containing 20 mM KCl from astrocytoma cells expressing the indicated channels (holding potential of −80 mV). (**f**) WT (black circles) and R18Q (grey circles) current densities elicited from each cell using the experimental protocol described in panel **e** which were subsequently averaged and depicted (mean ± SEM; *p < 0.05).

**Figure 2:**
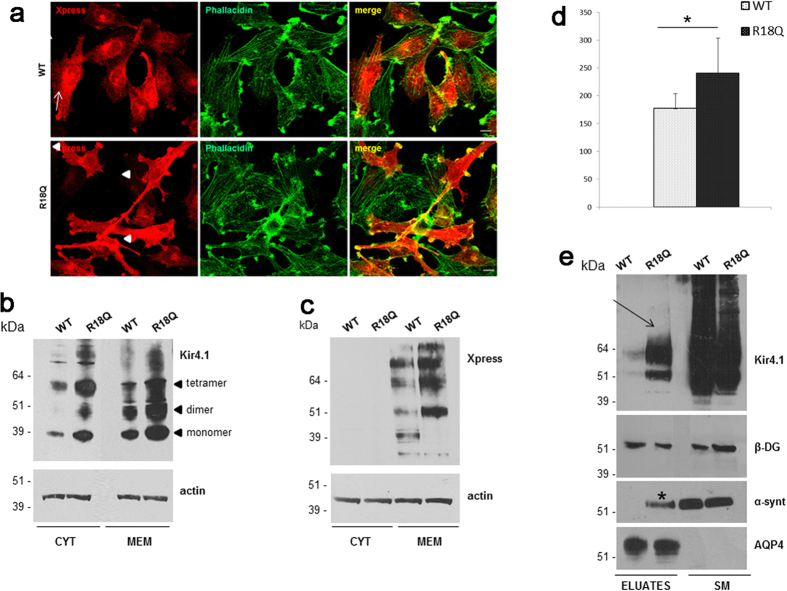
WT and mutated Kir4.1 expression and distribution in U251 astrocytoma cells. (**a**) Co-immunofluorescence staining of Kir4.1 WT and R18Q expressing cells using Anti-Xpress epitope Ab to stain recombinant Kir4.1 (red) and phallacidin to stain actin filaments (green) shows that WT channels are mostly localized in the cytoplasm, and at plasma membrane in a low percentage of cells (top panels, arrows), while R18Q mutant channels are mainly distributed along cell membranes, filopodia-like structures and cell-cell contacts (bottom panels, arrowheads), and partially co-localizes with actin, in the majority of cells (70%). (**b,c**) Western Blot (WB) analysis of total membrane and cytosolic protein extracts of astrocytoma cells expressing WT and R18Q Kir4.1, probed with anti Kir4.1 Ab (**b**) and Anti-Xpress epitope tag Ab (**c**) revealed that R18Q is more abundantly expressed in the cytoplasm (CYT) and particularly in the total membrane protein fraction (MEM), than the WT protein. Anti-Xpress epitope tag Ab does not detect Kir4.1 protein in the cytoplasm. Arrowheads on the right of panel b highlight the monomeric and oligomeric forms of the Kir4.1 channel. Actin is used as loading control. Molecular weight markers are on the left (kDa). (**d**) Densitometric analysis of recombinant Kir4.1 bands derived from total membrane protein extracts from WT or R18Q Kir4.1 expressing cells, detected by anti-Xpress Ab and normalized to the corresponding actin value (mean ± SEM, expressed as arbitrary units; *P < 0.05 from three independent experiments). (**e**) WB of total cell proteins (SM) and enriched fraction of plasma membrane proteins after biotinylation experiments (Eluates) probed with anti–Kir4.1 Ab. Higher amount of R18Q Kir4.1 is expressed at the plasma membrane (arrow) when compared to Kir4.1 WT. Among Kir4.1 associated proteins α-syntrophin (α-synt) is found at plasma membrane of Kir4.1 R18Q mutant expressing cells (asterisk) but not in WT expressing cells. No differences are observed in β-dystroglycan (β-DG) and AQP4 association to astrocytoma plasma membrane. One representative experiment out of two performed with the same results has been shown.

We have previously shown that the R18Q mRNA injection into *X. laevis* oocytes resulted in larger Kir4.1 currents than the WT, although the effects of this mutation were not assessed at the biochemical level and in a more physiological setting^2^. In newly generated U251MG astrocytoma cell lines stably expressing WT or R18Q channels, patch-clamp recordings showed that the averaged current densities of cells expressing the mutant channels were markedly larger than WT at both more positive and negative potentials than the K^+^ equilibrium potential (E_K_) (Fig. 1a–c). However, as shown in the dot plot of Fig. 1c (*inset*), the current density of cells expressing the mutant channel displayed a variability much higher than that observed in WT cells. The cell resting potential was on average ~4 mV more negative for R18Q compared to WT (Fig. 1d). To test the responsiveness of U251 cells expressing Kir4.1 channels to abrupt elevations of extracellular K^+^, as it may occur in tripartite synapses during intense neuronal activity, 300 ms puffs of K^+^ (20 mM) were delivered onto cells while recording whole-cell currents (Vh–80mV). These assessments showed that the K^+^-induced inward current density, recorded from R18Q expressing cells, was larger than the WT (Fig. 1e,f). Overall, these results indicated that the R18Q mutation increases Kir4.1 currents regardless of the expression system used to establish this effect (U251MG cells *vs X. laevis* oocytes) and enhances the influx of K^+^ upon increments of the concentration of this ion in the extracellular compartment. To investigate the hypothesis that a different distribution of WT and mutated channels in astrocytoma cells could account for the effects described above, we carried out immunofluorescence and western blot (WB) experiments. We found that untransfected U251 cells expressed low levels of endogenous Kir4.1 channels that were almost exclusively localized in the cytoplasm (Supplementary Fig. S1a), as previously observed in other glioma cell lines^4^. Immunostaining of U251 cells expressing recombinant Kir4.1 indicated that WT channels were mostly localized in the cytoplasm, both within perinuclear areas and along cell extensions and scarcely at plasma membrane (Fig. 2a, arrows, *top panels*). By contrast, the majority of cells (~70%) expressing R18Q mutant showed channels abundantly distributed along cell membranes, particularly in cell processes, filopodia-like structures and cell-cell contacts, where Kir4.1 partially co-localizes with actin (Fig. 2a, arrowheads *bottom panels*). RT-PCR analysis indicated that WT and R18Q cells expressed comparable levels of recombinant gene mRNAs, suggesting no differences in the infection levels between the two cell populations (Supplementary Fig. S1b). Consistently, WB analyses of protein derived from cytosol and total membranes (intracellular and plasma membrane) using anti-Kir4.1 Ab (Fig. 2b) or anti-epitope-tag Ab (Anti-Xpress) for the detection of recombinant channels (Fig. 2c,d) showed that R18Q channels were expressed more abundantly than WT protein in the membrane fraction. Biotinylation experiments and WB analyses were then performed to specifically evaluate WT and mutant Kir4.1 protein expression at plasma membrane (*see methods*). Higher levels of mutant Kir4.1 were found at the cell surface when compared with WT protein (Fig. 2e). Interestingly, α-*syntrophin* (α-synt), a protein belonging to the dystrophin-associated glycoprotein complex (DGC) which anchors Kir4.1 to glial cell membranes, was detected at much higher levels in cell eluates containing mutant Kir4.1 protein when compared to WT. No differences were seen in the amounts of *β-dystroglycan* (*β*-DG) and *aquaporin-4* (AQP4), two additional components of DGC bound to α-synt in glial cell plasma membranes (Fig. 2e). Co-immunostaining with α-syntrophin and Kir4.1 Abs indicated that the proteins co-localized in cells expressing the R18Q mutant in a higher percentage with respect to cells expressing the WT channel (Supplementary Fig. S2).

#### The p.R18Q mutation does not affect protein stability, membrane compartmentalization and molecular interactions

**Figure 3:**
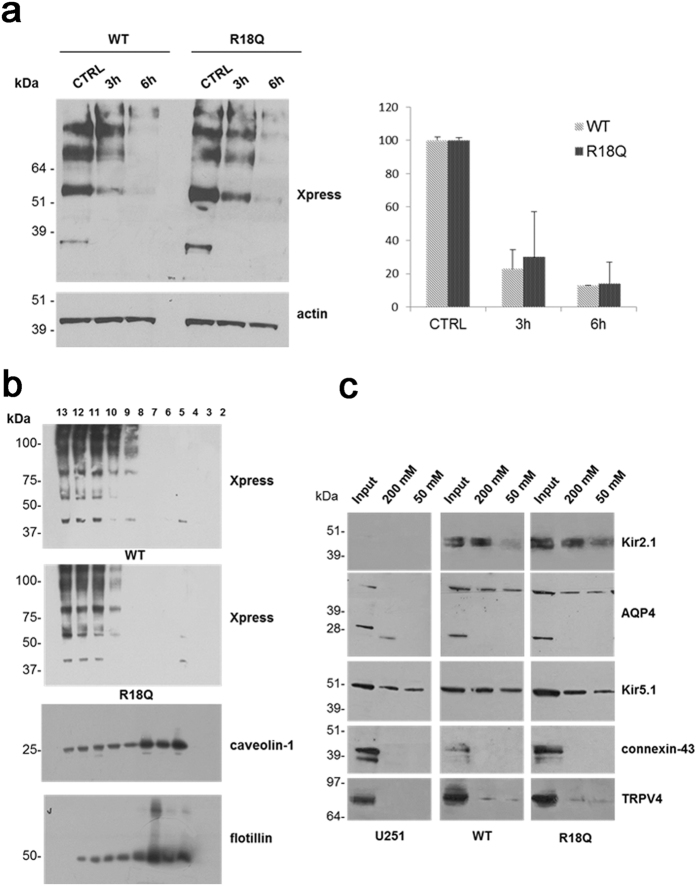
Degradation kinetics, membrane compartmentalization and molecular interactions of WT and R18Q Kir4.1. (**a**) Left panel: WB analysis of protein extracts obtained from cells expressing WT and R18Q Kir4.1 channels treated with the protein synthesis inhibitor cycloheximide (CHX) for 3 and 6 h to inhibit protein synthesis and evaluate protein degradation kinetic. Anti-Xpress epitope Ab was used to detect WT and mutant Kir4.1. Actin is used as loading control. Molecular weight markers are on the left (kDa). Right panel: densitometric analysis of WT or R18Q Kir4.1 protein bands after CHX treatment, normalized to the corresponding untreated controls, indicates no significant differences in the degradation kinetics between WT and R18Q Kir4.1 proteins. Data are expressed as mean ± SEM from three independent experiments. (**b**) WB analysis of cholesterol-rich (Triton insoluble fractions: 4–7) and cholesterol-poor membrane fractions (Triton soluble fractions: 10–13) obtained from WT or R18Q Kir4.1 expressing cells shows that WT and mutant Kir4.1 channels are both distributed in cholesterol-poor membrane fractions. Caveolin-1 and flotillin identify the caveolar raft fractions in cells expressing Kir4.1 WT. Molecular weight markers are on the left (kDa). (**c**) WB analysis of Kir4.1 channel interactors selected by Histidine (His) affinity chromatography. Eluates derived from astrocytoma cells infected with the empty vector (U251) were used as controls for unspecific binding to NiNTA-resin. The Input lane indicates the total protein extracts before His pull-down assay and 200 mM and 50 mM the proteins eluted from NiNTA-resin using imidazole (50, 200 mM, respectively). Kir2.1, aquaporin-4 (AQP4), Kir5.1, TRPV4, but not connexin-43 were found among the proteins co-eluted with Kir4.1. No difference was observed between WT and R18Q Kir4.1 interactors. One representative experiment out of four is shown. Molecular weight markers are indicated on the left (kDa).

The enhancement of membrane expression and current density, induced by p.R18Q in the presence of normal mRNA expression, raised the possibility that these effects could result from increased stabilization of the mutant protein at the plasma membrane and alterations in protein degradation kinetics, similar to that observed for Kir2.1 channels carrying a mutation linked to AEP^5^. To verify this possibility, cells expressing WT and R18Q channels were treated for 3 and 6 h with cycloheximide (CHX) to inhibit protein synthesis. Subsequent WB analysis revealed no statistical differences in the degradation kinetics of WT and R18Q proteins (Fig. 3a). Since Kir4.1 channels require cholesterol for their function^6^,^7^, and their distribution in cholesterol-enriched membrane rafts can modulate Kir4.1 channel activity^6^, we tested whether p.R18Q mutation could influence channel compartmentalization at astrocyte plasma membrane, as observed for the Kir2.1 mutant associated to AEP^5^. Analysis of Kir4.1 protein distribution in sucrose gradient-isolated cholesterol-rich or poor membrane fractions indicated that both WT and mutant channels were equally distributed in cholesterol-poor membrane fractions (Fig. 3b), excluding that p.R18Q could alter compartmentalization of Kir4.1 channels in lipid raft. We also tested whether p.R18Q mutation could influence Kir4.1 interactions with proteins known to bind directly Kir4.1 channel or associate indirectly to it via α-synt binding in glial cells, such as Kir2.1, Kir5.1, AQP4, TRPV4 and connexin-43^6^,^8^,^9^,^10^,^11^. We found the co-presence of Kir2.1, AQP4, Kir5.1, TRPV4, but not connexin-43, with Kir4.1 in the protein eluates, without significant differences between cells expressing WT and mutant channels (Fig. 3c).

#### Functional characterization of p.R348H and p.R271C variants

**Figure 4:**
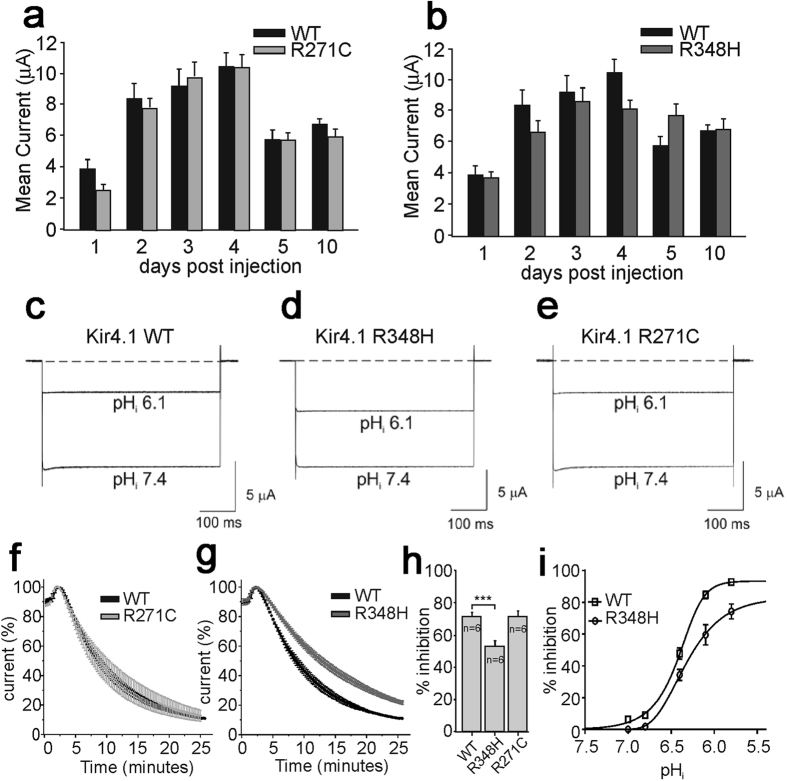
WT, R271C and R348H channels expression pattern and pH-gating in X.laevis oocytes. Mean current amplitudes during 10 days post mRNA injection: (**a**) WT *vs* R271C, and (**b**) WT *vs* R348H. Sample current traces recorded at pHi 7.4 and 6.1 from WT (**c**), R348H (**d**), and R271C (**e**) expressing oocytes. Time course of current inhibition (%) of R271C *vs* WT (**f**), and R348H *vs* WT (**g**) upon K^+^-acetate pHi acidification from 7.4 to 6.1. All traces were recorded at −100 mV from a holding potential −10 mV. (**h**) Bar graph of pHi 6.1-induced current inhibition (%) of the indicated channels. (**i**) pHi/inhibition relationships for WT (square) *vs* R348H (circles) assessed by using *X. laevis oocytes* and the K^+^-acetate pHi acidification method^12^,^13^,^14^.

**Figure 5:**
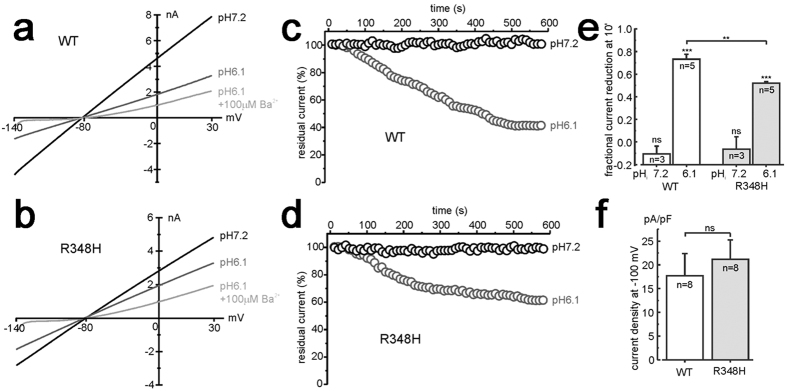
p*H*-gating of R348H channels expressed in astrocyte-like cells. Sample current traces for WT (**a**) and R348H (**b**) channel expressed in U251 cells, and recorded in the whole-cell configuration by applying voltage ramps from −140 to +30 mV (holding potential of −80 mV), immediately after the establishment of the whole-cell configuration (pHi7.2), after 10 min (pHi6.1), and following external BaCl_2_ application (pHi6.1 + 100 μM Ba^2+^). Both cells were recorded using an intracellular pipette solution having a pH of 6.1. (**c**,**d**) Representative time courses of WT (**c**) and R348H (**d**) currents recorded at −100 mV (normalized to the current recorded immediately after the establishment of the whole-cell configuration), using a pipette solution with pH of either 7.2 (black circles) or 6.1 (grey circles). (**e**) Plot of the mean fractional WT and R348H current inhibition by an intracellular pH of either 7.2 or 6.1. The fractional current reduction was estimated at −100 mV and calculated as 1− (I_10 min_/I_0 min_), in experiments similar to those shown in panels **a**–**d**. (**f**) Plot of the mean WT and R348H current density at −100 mV of applied potential.

Using two-electrode voltage-clamp (TEVC) and patch-clamp recordings from *X. laevis* oocytes injected with equal amounts of WT or mutated Kir 4.1 mRNAs, we observed that both p.R348H and p.R271C mutant channels yielded currents with macroscopic kinetics equal to WT. Furthermore, the current/voltage relationships for both mutant channels showed current amplitudes and inward-rectification similar to WT. Patch-clamp single-channel recording showed similar conductance for WT, R271C and R348H channels which were 24.1 ± 0.3 pS, 25.3 ± 0.6 pS and 25.7 ± 0.4 pS (n = 5; p > 0.05), respectively. To assess whether mutations interfere with physiological channel turnover, current amplitude was monitored during the 10 days following mRNA injection into oocytes. Also in this case, the trend did not differ between WT and mutants (Fig. 4a,b). We therefore tested the effects of intracellular acidification on the WT and mutant channels in oocytes (Fig. 4c–h) because it has been established that Kir4.1 channels are inhibited by intracellular acidification and WT current amplitudes decrease when pH_i_ is lowered to 6.1^12^,^13^,^14^. We found that whilst the p.R271C did not affect the pHi sensitivity of the channel (Fig. 4e,f,h), the p.R348H variant reduced significantly the acidification-induced current inhibition at plateau (Fig. 4d,g,h). To further investigate the pH_i_ sensitivity of WT and R348H channels, several pH_i_ values were tested. Figure 4i shows that the R348H mutation caused a right-shift of the pHi/inhibition relationship compared to WT. The WT and R348H current sensitivity to intracellular acidification was also assessed using U251 cells stably expressing these channel types. Currents were recorded at −100 mV using the whole-cell patch-clamp configuration and pipettes were filled with an intracellular solution having pH values of either 7.2 or 6.1. Both WT and R348H current amplitudes recorded at pH 7.2 were highly stable over time, after the establishment of the whole-cell configuration (Fig. 5a–d). Remarkably, the R348H currents were inhibited significantly less than the WT when a pipette solution at pH 6.1 was used (Fig. 5a–e). These recordings showed that the mean current densities for WT and R348H channels were similar also in U251MG cells (Fig. 5f), confirming the results obtained by using *X. laevis* oocytes (Fig. 4b). Collectively and consistently, these data showed that the R348H mutant channel displays a reduced sensitivity to intracellular acidification.

To assess whether the p.R348H and p.R271C variants influence Kir4.1 channel trafficking, we tested their possible alterations in expression and intracellular distribution in U251 cells. WB analysis (Supplementary Fig. S3a) and co-immunostainings (Supplementary Fig. S3b) indicated no differences among WT and mutant channels. Similarly, no significant differences were observed in the interactions of WT and mutant Kir4.1 with some of the main molecular interactors found in astrocytes (Supplementary Fig. S4). Overall, our data suggest that the p.R271C variant is benign, whereas the R348H variant most likely leads to GoF of Kir4.1 channels by reducing the pHi-sensitivity of the channel.

Based on crystal structure data, we finally generated a 3D-homology model of a Kir4.1 channel (See Supplementary Methods). The position of the Arg-18 residue in the N-terminal region could not be determined due to lack of structural data. However, the model suggests that the N-terminal, where the R18Q mutation is located, likely interacts with the C-terminal (Supplementary Fig. S5a). The analysis of this modelling also indicated that the R348H mutation is located in the C-terminal region of the cytoplasmic domain of Kir4.1 channel (Supplementary Fig. S5b). A 3D-homology model for the R271C channel was also generated, showing that this mutation affects an arginine residue located in the intracellular domain and pointing towards the bulk solvent (Supplementary Fig. S6a,b).

#### *In vivo* modelling of Kir4.1 mutations in zebrafish (D. rerio)

**Figure 6:**
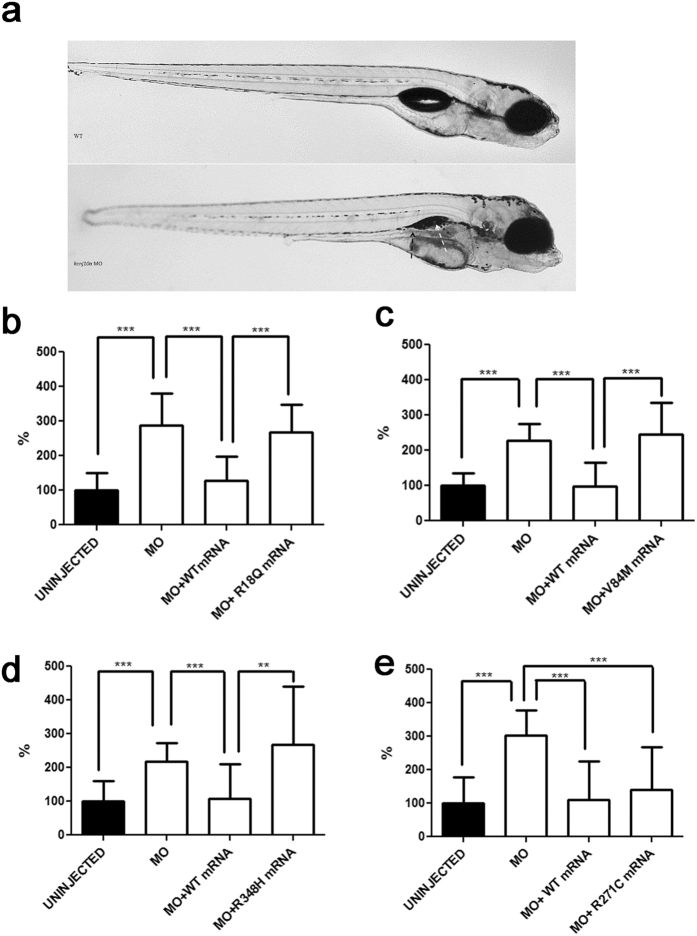
*In vivo* modelling of Kir4.1 mutations in zebrafish. (**a**) Transient *kcnj10a* knockdown zebrafish show macroscopic abnormalities in organ development compared to wild-type (WT). In morphant embryos, the pronephric duct is visible because it is dilated (black arrow); the swim bladder is not visible (white arrow). (**b–e**) Number of spontaneous tail flicks (registration time 30sec) seen in WT and mutant 30 hpf embryos. Values are expressed as percent of flicks counted in uninjected embryos. Embryos injected with MO (**b–e**), and with equimolar amount of MO and either R18Q (**b**), V84M (**c**), and R348H (**d**) mRNA show an increased rate of spontaneous contractions, compared to uninjected embryos (**b–e**), and to either MO+WT (**b–e**) and MO+R271C (**e**) mRNA injected embryos. **p < 0.01; ***p < 0.001.

Using an established methodology^15^, transient knockdown of *kcnj10a* through morpholino (MO)-based technology led to macroscopic abnormalities in organ development (Fig. 6a), as well as 2- to 3-fold increases of spontaneous tail flicks in 30 hpf morphants (Fig. 6b–e). This pathological phenotype is consistent with data in morphants observed by others and possibly relates to neuronal hyperexcitability underlying epilepsy^15^. To test *in vivo* the effects of human Kir4.1 variants detected in AEP children on the disease phenotype seen in zebrafish, we also co-injected embryos with MO and equimolar amounts of either WT or mutated human *KCNJ10* mRNA. While the WT and the R271C mRNA could rescue the number of spontaneous tail flicks to basal levels, the human mRNA harbouring each of R18Q, V84M and R348H could not complement the diseased phenotype (Fig. 6b–e). A similarly abnormal locomotor behaviour was observed in WT embryos overexpressing the R18Q mutation, either alone or co-injected with the WT human mRNA, suggesting a GoF effect of the mutant allele (Supplementary Fig. S7).

## Discussion

The inwardly-rectifying K^+^ channels Kir4.1 are strongly expressed in the brain, mainly in the cortex, thalamus, hippocampus, and brainstem^16^,^17^, where they regulate the astrocyte resting membrane potential, differentiation and K^+^ homeostasis. The polarized transport of K^+^ from regions of high [K^+^]_o_, resulting from synaptic excitation, to those of low [K^+^]_o_ is essential to maintain the ionic and osmotic environment in the extracellular space, and to facilitate glutamate reuptake^18^,^19^,^20^,^21^,^22^. Although primarily expressed in astrocytes^23^, Kir4.1 channels are key regulators of neuronal excitability^24^, especially during high activity^25^, and of astroglial-dependent synaptic functions, such as hippocampal short-term plasticity^26^. When these mechanisms are impaired, they may impact on cognitive development^27^, as well as motor function and susceptibility to seizures^28^. Conditional knock-out mice lacking Kir4.1 in astrocytes manifest abnormal brain development, ataxia, seizures, and early postnatal death, thus supporting the crucial role of the channel for normal brain function^19^. *Loss-of-function* (LoF) defects of Kir4.1 (*KCNJ10*) have been linked to the autosomal recessive SeSAME/EAST Syndrome (MIM 612780)^29^,^30^, a condition encompassing clinical features such as sensorineural deafness, renal salt-losing tubulopathy, early onset seizures, ataxia, and severe intellectual disability. When modelled in zebrafish, LoF of Kir4.1 channels recapitulate the human disease in terms of epilepsy and behaviour^15^. Severe motor impairment (ataxia and myokymia) and seizures, but not hearing loss or renal impairment, are also the clinical manifestations seen in Russell Terriers dogs harbouring LoF homozygous mutations in *KCNJ10*^31^. Seizures, intellectual disability, and ASD, the features that define AEP, represent however the main clinical presentation of patients with GoF mutations of *KCNJ10*^2^, confirming that Kir4.1 dysfunction likely increases the susceptibility to abnormal behaviour typical of ASD, in addition to epilepsy and cognitive impairment^32^,^33^.

With this in mind, we have collected a broader sample of ASD patients, with or without epilepsy, in order to screen *KCNJ10* and perform genotype-phenotype correlations. We detected mutations (p.R18Q, p.V84M, and p.R348H) in nine children (5.1%), confirming that Kir4.1 defects are not an exceptionally rare event in AEP. Clinical investigations in our group of patients showed that gene variants were significantly associated with infantile spasms, with a relatively good outcome, lower frequency of stereotyped behaviours, and augmented dysregulation of sensory processing profiles, when compared to non-mutation carriers (Table 2). Thus, these clinical features seem to define a possible Kir4.1-related endophenotype among the multitude of polymorphic conditions that characterize epilepsies and ASD^34^,^35^. We also observed that the p.R18Q variant was the most frequent mutation in AEP, and that it causes GoF defects by promoting the trafficking of Kir4.1 channels at plasma membranes and enhancing K^+^ fluxes across cell membrane, that largely substantiate the data we had reported earlier using *X. laevis* oocytes^2^.

The strong role of astroglial Kir4.1 channels for K^+^ buffering is largely due to their high open probability at rest, and their increased conductance upon build-up of extracellular K^+^ concentrations^25^,^36^ that usually occurs in the brain during high neuronal activity. Immunofluorescence staining showed that the p.R18Q mutation promotes Kir4.1 channel expression particularly at the end-feet, the structures where K^+^ siphoning occurs. In addition, our patch-clamp recordings indicated that astrocyte-like cells expressing the p.R18Q possess an even increased ability to uptake extracellular K^+^ions compared to WT, when exposed to higher extracellular K^+^ concentration. A precise distribution of the K^+^ channels, accumulating at appropriate density in discrete subdomains of glial cell membranes, is a requirement for proper astrocyte-mediated K^+^ buffering activity, an essential process for the maintenance of optimal K^+^ concentrations in the extracellular environment, and consequently for correct synaptic functionality^37^,^38^. An altered homeostasis at the tripartite synapse, induced by GoF defects of Kir4.1 channels in astrocytes, may represent therefore a possible mechanistic hypothesis linking our identified allelic variations with the increased susceptibility to seizures, as well as the behavioural and cognitive impairment.

Recent studies demonstrated that an AEP-related GoF mutation in Kir2.1 channels increased their stabilization at the astrocytoma plasma membrane by hampering channel compartmentalization in membrane lipid rafts and the consequent caveolar-mediated endocytosis and proteasome degradation^5^. Differently from those findings, Kir4.1 mutations do not seem to influence channel degradation kinetics and lipid-raft distribution. Conversely, R18Q mutants seem to influence channel anchoring at plasma membrane. Biotinylation assays in astrocytoma plasma membrane revealed an increased expression in this compartment of the α-synt protein in cells expressing the mutant but not the WT channels. Accordingly, co-immunostainings indicated that co-localization of α-synt and Kir4.1 was higher in cells expressing the R18Q mutant than in cells expressing the WT Kir4.1. By binding directly to Kir4.1 intracellular COOH terminal, α-synt anchors channels to astrocyte plasma membrane keeping them in close contact to DGC associated proteins, including AQP4^37^. The localization of the R18 residue in the intracellular NH_2_ terminal of the Kir4.1 protein leads us to speculate that the mutation might influence the binding of α-synt at the COOH terminal by affecting the complex tridimensional molecular assembly characterizing the channel *in vivo*. Indeed, functional Kir4.1 channels are assembled into homo-tetramers where the NH_2_ terminals lie between adjacent COOH residues and contribute to the formation of the interface between subunits (Supplementary Fig. S5)^2^,^22^. Moreover, the effects of p.R18Q mutation on the molecular interactions between Kir4.1 and α-synt seem to be specific, since no differences have been observed in the interactions with other proteins that, directly or indirectly via DGC, associate with Kir4.1 (i.e., Kir2.1, AQP4, Kir5.1, TRPV4) (Fig. 3). It is our opinion that the reinforced interaction of the Kir4.1 molecules to α-synt, induced by the mutation, could therefore mechanistically explain the increased amount of channels observed at astrocyte plasma membrane, and the consequent GoF of the R18Q channel.

The much rarer p.R348H, observed in a single child with AEP, was more difficult to reconcile with a GoF mechanism, biochemically, though it affected embryo locomotion and tail flicks behaving as the other two severe Kir4.1 variants (p.R18Q and p.V84M) when co-injected in zebrafish morphants (see Fig. 6d). TEVC and patch-clamp investigations showed that the p.R348H was less sensitive to intracellular acidification than WT. Glial cells undergo substantial intracellular pH changes, for instance during neuronal activity, in the presence of high levels of extracellular neurotransmitters^39^,^40^,^41^, or upon glutamate uptake^42^,^43^. Intracellular acidosis has also been associated with glutamate release from astrocytes^44^. Thus, the differential pH sensitivity of Kir4.1 channels carrying the R348H may result in significant functional consequences for the brain under critical conditions such as intense neuronal activity or build-up of neurotransmitters in the synaptic cleft. Noteworthy, an altered pH-gating of Kir channels has been also proposed as a new pathomechanism underlying antenatal Bartter syndrome type 2 (BS). Indeed, several LoF mutations in *KCNJ1* (Kir1.1) have been associated with BS through a shift of pH sensitivity of Kir1.1 channels to more alkaline values^45^. Similarly, we might postulate a GoF effect due to altered pH-gating of Kir4.1 channels as a possible disease mechanism contributing to ASD, epilepsy, or to their concurrence. On the other hand, our investigations (both in cellular expression systems and in zebrafish) casted further doubts on a meaningful role for the p.R271C variant detected in 10 AEP children in keeping with previous results^46^. On the whole, our studies did substantiate the disease significance (though minor) of variants in Kir4.1 in ASD and comorbid epilepsy and offered new possible molecular therapeutic targets in neurodevelopmental disorders.

A last consideration emerged from our preliminary observations in zebrafish. When compared to data gathered by others in the SeSAME/EAST syndrome, we observed that GoF and LoF mutations in Kir4.1 appear to lead to a similar breakdown of complex homeostatic mechanisms that, through fine regulation of neuronal excitability and synaptic activity, control the physiological development of crucial neurological functions. A possible explanation could be, indeed, that the mutant mRNA would result in a neomorphic protein with “toxic” GoF effect, i.e. due to the ectopic cellular expression of the mutant channels. It is hard to know, however, why the LoF phenotypes in the SeSAME/EAST syndrome are partially different compared to our children (*i.e.*, a more severe ataxic disorder, or the presence of hearing and renal involvements not obvious in AEP). This could in part be explained by a milder functional impact of heterozygous gene variants (seen in AEP) compared to homozygous mutations seen in the SeSAME/EAST syndrome or that GoF and LoF mechanisms may affect the human neurodevelopmental process in a different way and with different degrees of methylation-dependent transcriptional regulation, known to affect functional Kir4.1 channels during late foetal and early post-gestational life^47^,^48^, a time period where epigenetic processes critically fine-tune the neurodevelopmental program^49^. Regardless of these considerations, yet deserving further studies, our findings offer new insight on the complex machinery by which astrocytes might regulate neuronal excitability and synaptic function in ASD and seizures, and promote future investigations on the mechanisms by which the dysfunctional channels, or their molecular interactors, may be targeted by customized pharmacological treatments.

## Materials and Methods

### Patients and measures

A sample of 175 consecutive individuals with idiopathic ASD [24 F (13.7%) and 151 M (86.3%), aged 2.0 to 20.8 years (7.5 mean ± 4.1 SD)], was divided in two groups: I) AEP (ASD with seizures and/or EEG abnormalities; 137/175, 78.3%) and II) “simplex” forms of ASD (with no history of seizures nor EEG abnormalities; 38/175, 21.7%). Individuals with non-idiopathic autism or symptomatic epilepsy had been excluded before enrolment. Phenotype characterization was carried out through clinical assessment, EEG study, and brain magnetic resonance imaging, as previously reported^50^,^51^,^52^. A family history for epilepsy or ASD was investigated up to the 4th degree of kinship. The presence of a parental BAP (broader autism phenotype)^53^ was assessed through the semi-structured interview BPASS^3^.

*Statistical analyses*, using the IBM© SPSS software version 20, were conducted in order to draw genotype-phenotype correlations. We used t-test for continuous variables, and the Chi-squared test to analyse categorical variables. A correspondence analysis was used to decompose the significant Chi-squared and reduce variables dimensions. Significance level was set at p < 0.05. Statistically significant data are indicated by asterisks in the tables.

### Genetic analyses

Total genomic DNA from peripheral blood was obtained from patients using standard purification protocols. Screening of variants in *KCNJ10* was performed with Sanger sequencing as described^2^. Kir4.1 variants were also screened in a large cohort of in-house pooled control DNA samples (280 Italian individuals), and in 248 additional DNA samples (NA16129, NA1660, and NA16601) of different ethnicities from the Coriell Institute for Medical Research (Camden, NJ).

### Functional assays *in vitro*

#### Generation and treatments of U251 astrocytoma cell lines expressing His-tagged Kir4.1 WT or carrying p.R18Q, p.R348H, or p.R271C variants

Astrocytoma U251MG cell lines were obtained from the American Type Culture Collection (Rockville, MD) and grown as previously described^5^. We generated and stably overexpressed His-tagged Kir4.1 either WT or carrying each of the three *KCNJ10* variants investigated in this study (R18Q, R348H, R271C) in astrocytoma U251 cell lines using methodologies previously reported^5^. For cell treatments, astrocytoma cell lines were plated in 100-mm diameter dishes and treated for different time lengths (3h, 6h) with CHX (100 μg/ml, Sigma).

#### Immunofluorescence and confocal microscopy analyses

Cells were grown subconfluent on polylysine-coated coverslips and fixed and stained using the following Abs: affinity purified anti-Xpress mAb (1:100, Life Technologies), anti-Kir4.1 pAb (1:50, Alomone Labs) and anti-syntrophin mAb (1: 50, Millipore) as primary Ab and TRITC-conjugated goat anti-mouse IgG Ab (1:100, Jackson Immunoresearch Laboratories), biotinylated goat anti-rabbit IgG H + L Ab (4.3 μg/ml; Jackson Immunoresearch Laboratories, West Grove, PA) or Alexa Fluor 488 goat antimouse IgG Ab (1:300, Invitrogen, Milan, Italy) as secondary Ab^5^. Actin filaments were stained using N-(7-nitrobenz-2-oxa-1,3-diazol-4-yl) (NBD)-phallacidin (1:30, Life Technologies). Coverslips were washed, sealed in in ProLong Gold antifade reagent with 4,6-diamidino-2-phenylindole (Invitrogen) and analysed with a laser scanning confocal microscope (LSM 5 Pascal, Carl Zeiss, Jena, Germany).

#### Purification of histidine-tagged proteins by affinity chromatography

Lysates obtained from two T175 cm^2^ flasks of confluent astrocytoma cell lines stably expressing His-tagged WT or mutated Kir4.1 and mock infected control (U251) cells were used to perform the enrichment and purification of His-tagged proteins following already described procedures^8^. Fractions containing eluted protein were analysed by WB, as described below.

#### Detergent-resistant microdomain (DRM/lipid rafts) preparation by sucrose gradients

DRMs from cultured astrocytoma cell lines expressing His-tagged WT and mutated Kir4.1 were prepared essentially as described previously^54^. DRM fractions were visible as a light-scattering band migrating at approximately 20% sucrose (fractions 4, 5, 6). Samples were precipitated overnight with acetone (1:4 v/v) and proteins analysed by SDS–PAGE and WB, as described below.

#### Protein extract preparation and Western blotting

Cytosolic, membrane fractions and total protein extracts from cultured astrocytoma cell lines were obtained as previously described^8^,^54^. Equal amounts of proteins (30 μg) were resolved on SDS-PAGE using gradient (4–12%) precasted gels (Invitrogen), and transferred onto a nitrocellulose membrane. Nitrocellulose membranes were blotted overnight at 4 °C using the following primary Abs diluted in PBS/3% BSA: anti-Kir4.1 pAb (1:800, Alomone Labs), anti-Xpress mAb (1:2500, Life Technologies), anti-β-DG mAb (1:25, Novocastra Lab), anti-caveolin-1 pAb (1:1000, Santa Cruz Biotecnology), anti-Kir5.1 pAb (1:500, Abcam), anti-flotillin mAb (1:1000, BD Transduction Laboratories), anti-syntrophin mAb (1:200, MA-1-745, Affinity BioReagents, CO), anti-TRPV4 pAb (1:200, Alomone Labs), anti-connexin-43 mAb (1:250, BD Transduction Laboratories), anti-Kir2.1 pAb (1:250, Alomone Labs), anti-aquaporin-4 mAb (1:200, Santa Cruz Biotecnology), anti-actin mAb (1:2000, Santa Cruz Biotecnology). As secondary Abs we used peroxidase conjugated anti-mouse or anti-rabbit Abs (1:10000; Thermo Scientific, MO), for 1 hour at room temperature (RT). Immunoreactive bands were visualized using an enhanced chemiluminescence reagent (Thermo Scientific), according to the manufacturer’s instructions and exposed on X-ray films.

#### Cell surface protein biotinylation

Cell surface protein biotinylation assay was performed using a commercially available biotinylation kit (Thermo Scientific), following previously described procedures^55^. Fractions containing eluted proteins were analysed by WB, as described above.

#### Expression of Kir4.1 channels in X. laevis oocytes

The human Kir4.1 constructs were generated as previously described^2^. Stages IV–V *X. laevis* oocytes were isolated, injected with 50 nl mRNAs and stored at 16 °C in fresh ND96 medium containing (in mmol/L): NaCl 96, KCl 2, MgCl_2_ 1, CaCl_2_ 1.8, Hepes 5, gentamicin 50 μg/ml (Sigma, Italy). mRNA concentrations were quantified by electrophoresis and ethidium bromide staining and by spectrophotometric analysis. Equal amount of either WT or mutant mRNAs were then microinjected into oocytes^2^.

#### Electrophysiology

TEVC recordings were performed from oocytes at RT (22 °C) and 1–10 days after injection by using a GeneClamp 500 amplifier (Axon Instruments, Foster City, CA) interfaced to a PC with an ITC-16 interface (Instrutech Corporation, Longmont, CO), as previously described^56^,^57^. Standard recording solution contained (in mmol/L): KCl 90, MgCl_2_ 3, Hepes 10 (pH 7.4). Recordings were filtered at 2 kHz and acquired at 5 kHz with Pulse software and analysed with either PulseFit (HEKA, Germany) or IGOR (WaveMetrics, Lake Oswego, OR)^58^. Intracellular acidification was achieved using a potassium acetate buffering system^12^,^59^. Only one pH/inhibition value per cell was determined in TEVC experiments as previously described^13^,^14^. To assess the effects of intracellular acidification we used a well-established potassium acetate buffering system able to modify the intracellular pH of oocytes^59^.

*Patch-clamp recordings* were performed in *X. laevis* oocytes using the cell-attached and inside-out configuration at 22 °C and an Axopatch 200B amplifier (Axon Instruments) as described^60^. Briefly, oocytes were bathed in a cytoplasmic solution containing (in mmol/L) KCl 120, CaCl_2_ 1, EGTA 11, Hepes 10, dithiothreitol 0.1 (pH 7.2). Recording electrodes were filled with a solution containing (in mmol/L) KCl 120, Hepes 10, CaCl_2_ 0.2 (pH 7.2) and had resistance of 3–8 MΩ. Channel activity was analysed with a TAC-TACFit program (Bruxton Co., Seattle, WA) and channel openings were visually inspected before being accepted (*event-by-event mode*). Recordings in U251MG cells were performed using an Axopatch 200B Amplifier (Axon Instruments), at RT as described^5^. The extracellular recording solution contained (in mmol/L) NaCl 140, KCl 5, CaCl_2_ 2, MgCl_2_ 2, MOPS 5, glucose 20, (pH 7.4 with NaOH). In experiments where cells were puffed with high extracellular K^+^, KCl was increased to 20 mM and NaCl was decreased to 125 mM. The micropipette solution contained (in mmol/L) KCl 155, EGTA-K 1, MOPS 5, MgCl_2_ 1 (pH 7.2 or 6.1, when indicated, with KOH). To show Kir4.1 specificity, 100 μmol/L BaCl_2_ was added to the bath solution to block the inward rectifying current.

All the data are presented as mean ± SEM. Two-tailed Student’s t-test was used to compare means and statistic level was set with significance at p < 0.05.

### Generation of *kcnj10a* morphant zebrafish and co-injection with human Kir4.1 variants

Zebrafish *kcnj10a*, the orthologue of human *KCNJ10*, has already been cloned, and experimental procedures to study gene expression (load and site), to generate morphant embryos, and to investigate the significance of human mutations have already been described elsewhere^15^. To transiently knockdown the Kir4.1 function in zebrafish, we adopted an already established protocol using MO (Gene Tools, USA) targeting the splice site (5-AATTGTGAGAGCTATACCTTGGCGA-3) of *kcnj10a*, diluted into RNAse free water, and injected into one- to two-cell stage embryos^15^. The embryos were raised at 28.5 °C. For rescue experiments, human WT and R18Q, V84M, R348H, and R271C cDNAs were cloned in a pCS2 + vector. The capped mRNA was obtained using mMESSAGE mMACHINE SP6 transcription Kit (Life technologies, Carlsbad, CA), and 50 pg of human WT or mutant *KCNJ10* cRNA were co-injected with 0.5 ng of *kcnj10a* MO.

#### Spontaneous contraction and rescue analyses in zebrafish larvae

To test the effects of human Kir4.1 mutations on embryonic phenotype, we injected one-cell stage eggs with both MO and equimolar amounts of either WT, or R271C polymorphism, or mutated human mRNA, and compared spontaneous contractions at 30 hpf in injected, uninjected and morphant embryos. A dominant-negative effect of the mutant allele was also investigated by injecting one-cell stage eggs with either the R18Q or WT human mRNA alone, or with equimolar amounts of WT + R18Q human mRNA, and by comparing the resulting phenotypes^61^. Locomotor activity (15 embryos for each group in triplicate) was recorded for 180 sec, and the number of complete tail contractions measured in 30 sec time frames. Video recordings were made with Leica M80 microscope with Nikon Digital Sight DS-Fi1 camera and the NIS Elements software package (Nikon, Nikon Corp., Europe), and a digital video camera using the CamStudio software. Tail flicks counting data were placed in Prism (GraphPad) to generate graphs and to perform statistical analyses (unpaired two-tailed Mann-Whitney *U*-test of all pairwise combinations).

### Ethics Statement

All study protocols were approved by the Research Ethics Committee of the IRCCS Stella Maris Foundation, Pisa (Italy). All patients or their parents signed an informed consent prior to the assessment, and agreed for their medical data to be used anonymously in the research. All clinical procedures were carried out in accordance with the approved guidelines.

Procedures involving *X. laevis* and their care were in accordance with the regulations of the Italian Animal Welfare Act and in agreement with the NIH Guide for the Care and Use of Laboratory Animals. All experimental protocols were approved by the OPBA (Organismo Preposto al Benessere degli Animali) licensing body of the University of Perugia (Italy). The minimal number of animals was used.

Experiments in *D. rerio* were performed at the Italian National Research Council (NRC)-zebrafish core facility, in accordance with, and under the supervision of, the Institutional Animal Care and Use Committee (IACUC) of the University of Pisa and the NRC. All experimental protocols were approved by the animal ethics committee of the University of Pisa-NRC-Fondazione Monasterio. Animals were always managed and injected according to the principles of Good Animal Practice as defined by the Italian animal welfare regulations. Every effort was made to minimize animal suffering and to use the minimum number of animals necessary to collect reliable scientific data.

## Additional Information

**How to cite this article**: Sicca, F. *et al*. *Gain-of-function* defects of astrocytic Kir4.1 channels in children with autism spectrum disorders and epilepsy. *Sci. Rep.* **6**, 34325; doi: 10.1038/srep34325 (2016).

## Supplementary Material

Supplementary Information

## Figures and Tables

**Table 1:** Clinical features of individuals with *KCNJ10* variants.

	R18Q 7/175 (4%)	R348H 1/175 (0.6%)	V84M^2^1/175 (0.6%)
Phenotype			
AEP (137, 78.3%)	5	1	1
“Simplex” ASD (38, 21.7%)	2	0	0
Inheritance			
Maternal	4/7 (57.1%)	—	1/1
Paternal	2/7 (28.6%)	1/1	—
Both	—	—	—
Unknown	1/7 (14.3%)	—	—
Gender			
Male	7/7 (100%)	—	1/1
Female	—	1/1	—
ASD diagnosis			
Autism	—	—	1/1
PDDNOS	7/7 (100%)	1/1	—
Asperger’s syndrome	—	—	—
Seizures			
Yes	3/7 (42.9%)	1/1	1/1
No	4/7 (57.1%)	—	—
Type of seizures			
Focal	—	—	1/1
Generalized	1/3 (33.3%)	—	—
Spasms	2/3 (66.7%)	1/1	—
EEG abnormalities			
Yes	5/7 (71.4%)	1/1	1/1
No	2/7 (28.6%)	—	—
EEG Site			
Anterior	3/5 (60%)	—	1/1
Posterior	—	—	—
Temporal	—	—	—
Multifocal/Diffuse	2/5 (40%)	1/1	—
Cognitive Development			
Normal-Borderline	1/7 (14.3%)	—	—
Mild-Moderate Delay	5/7 (71.4%)	—	—
Severe Delay	1/7 (14.3%)	1/1	1/1

**Table 2:** *Gain-of-function KCNJ10* variants (R18Q, R348H, V84M) vs WT.

	WT (n = 166)	*KCNJ10* Variants (n = 9)	Test (χ^2^)	Effect size (Φc)	P
History of Seizures					
Yes	58 (34.9%)	5 (55.6%)	1.575	0.095	0.210
No	108 (65.1%)	4 (44.4%)			
Type of Seizures					
Focal	37 (63.8%)	1 (20%)	11.131	0.420	0.004*
Generalized	16 (27.6%)	1 (20%)			
Spasms	5 (8.6%)	3 (60%)			
EEG abnormalities					
Yes	122 (77.7%)	7 (77.8%)	0.000	0.000	0.996
No	35 (22.3%)	2 (22.2%)			
Site of EEG abnormalities					
Anterior	41 (33.6%)	3 (42.9%)	2.947	0.151	0.400
Posterior	18 (14.8%)	0			
Temporal	18 (14.8%)	0			
Multifocal/Diffuse	45 (36.8%)	4 (57.1%)			
Type of EEG abnormalities					
Paroxysms	59 (48.4%)	4 (57.1%)	1.279	0.100	0.528
Focal slowing	19 (15.6%)	0			
Both	44 (36%)	3 (42.9%)			
Stereotyped Behaviours					
Yes	144 (87.8%)	3 (37.5%)	4.217	0.157	0.040**
No	20 (12.2%)	5 (62.5%)			
Regulation Disorder of Sensory Processing					
Yes	109 (66.9%)	9 (100%)	4.346	0.159	0.037***
No	54 (33.1%)	0			

WT = Wild type; χ^2^ = the Pearson chi-squared test; Φc = Cramer’s phi coefficient; *KCNJ10 variants were significantly associated with epileptic spasms; **KCNJ10 variants showed stereotyped behaviours less frequently than WT; ***KCNJ10 variants showed a Regulation Disorder of Sensory Processing more frequently than WT.
