# Supplementary material for: Gain-of-function defects of astrocytic Kir4.1 channels in children with autism spectrum disorders and epilepsy
Source: Sci Rep. 2016 Sep 28;6:34325. doi: 10.1038/srep34325 (PMC5039625; doi:10.1038/srep34325)

Gain-of-function **defects of astrocytic Kir4.1 channels in children with autism spectrum disorders and epilepsy**

Federico Sicca[1,3,+,*], Elena Ambrosini[2,+,*], Maria Marchese[3], Luigi Sforna[4], Ilenio Servettini[4], Giulia Valvo[1], Maria Stefania Brignone[2], Angela Lanciotti[2], Francesca Moro[3], Alessandro Grottesi[5], Luigi Catacuzzeno[6], Sara Baldini[1], Sonia Hasan[4], Maria Cristina D'Adamo[4,7], Fabio Franciolini[6], Paola Molinari[8], Filippo M. Santorelli[3], Mauro Pessia[4,7,*]

1. Clinical Neurophysiology Laboratory, Department of Developmental Neuroscience, IRCCS Fondazione Stella Maris, Via dei Giacinti 2, 56128 – Pisa, Italy
2. Department of Cell Biology and Neuroscience, Istituto Superiore di Sanità, Viale Regina Elena 299, 00161 – Rome, Italy
3. Molecular Medicine Laboratory, Department of Developmental Neuroscience, IRCCS Fondazione Stella Maris, Via dei Giacinti 2, 56128 – Pisa, Italy
4. School of Medicine, Section of Physiology & Biochemistry, Department of Experimental Medicine, Piazzale Gambuli, University of Perugia, 06132 – Perugia, Italy
5. SuperComputing Applications and Innovation – CINECA, Via dei Tizii, 6, 00185 – Roma, Italy
6. Department of Chemistry, Biology and Biotechnology, University of Perugia, Piazzale Gambuli, 06132 – Perugia, Italy
7. Faculty of Medicine, Department of Physiology & Biochemistry, University of Malta, MSD 2080 – Msida, Malta
8. Department of Pharmacology, Istituto Superiore di Sanità, Viale Regina Elena 299, 00161 – Rome, Italy

[+]These authors contributed equally to this work.

*** Correspondence to**: Federico Sicca, IRCCS Fondazione Stella Maris, Via dei Giacinti 2 - Calambrone, 56128 Pisa, Italy. Tel: +39 050886234; Fax: +39 050886247; email: federico.sicca@fsm.unipi.it; and Elena Ambrosini (elena.ambrosini@iss.it) and Mauro Pessia (mauro.pessia@unipg.it; mauro.pessia@um.edu.mt)

Supplementary Clinical Data

Summary of the main clinical features characterizing patients with GoF mutations of Kir4.1. The age given is at the time of the latest clinical observation

Patient 1

This was a 12.1-year-old boy with Autism Spectrum Disorder (ASD), epilepsy and mild intellectual disability. Absence seizures started when he was 8, and were controlled with valproate therapy. EEG recordings showed bilateral anterior paroxysms on awake and sleep, and diffuse 3Hz spike-and-wave complexes associated to clinical absences. The R18Q variant was inherited from his father, who displayed focal EEG abnormalities as well as subclinical manifestations of autism involving social motivation, expressiveness, and flexibility/range of interests, as assessed by the BPASS.

Patient 2 [ref2]

This 8.2-year-old boy was an identical twin with regressive-onset ASD, epilepsy, nystagmus and moderate intellectual disability. At the age of 7 months, he exhibited epileptic spasms that remitted under ACTH therapy. At 10 months, he still showed seizures that stopped two months later on valproate-topiramate therapy. His sleep EEG showed multifocal abnormalities. The R18Q variant was inherited from his mother, who showed obsessive-compulsive symptoms and mood disorder.

Patient 3 [ref2]

This boy, aged 8.2 years and carrying the R18Q variant, was the identical twin of Patient 2, and presented with similar features including regressive-onset ASD, epilepsy, nystagmus and mild cognitive disability. Epileptic spasms started at 7 months, and remitted under ACTH therapy at age 10 months. EEG recordings showed multifocal abnormalities during sleep.

Patient 4

This 12.3-year-old boy displayed ASD, EEG epileptic abnormalities (anterior paroxysms during sleep), and mild cognitive disability. The R18Q variant was inherited from his father.

Patient 5

This boy aged 8.2 years displayed ASD, EEG paroxysmal abnormalities and severe intellectual disability. EEG recordings showed anterior paroxysms on awake and sleep. The R18Q variant was inherited from his mother.

Patient 6

This 5.7-year-old boy displayed ASD, mild intellectual disability, and bilateral severe neurosensory deafness, treated with cochlear implant, due to a compound heterozygosity in the connexin-26 (*GJB2*) gene (35delG and I20T). At the latest follow-up, EEG recording was normal and he had no history of seizures (simplex ASD). The R18Q variant was inherited from his mother.

Patient 7

This 12-year-old boy, carrying the R18Q variant, presented with ASD, normal cognitive development, and no history of seizures or EEG abnormalities (simplex ASD). He had subclinical hypothyroidism, treated with levothyroxine. No family study was performed.

Patient 8

This was a 7.6 year-old girl with regressive ASD, epilepsy and severe intellectual disability. Epileptic spasms started when she was 4 month-old, and remitted without treatment two months later in connection with a febrile illness. EEG recordings showed multifocal abnormalities during sleep. She also displayed alternant exotropia and nystagmus. The

R348H variant was inherited from her father, who displayed a severe anxiety disorder with panic attacks, and focal abnormalities on EEG recordings.

Patient 9 [ref2]

This boy aged 14 is the first child of healthy, non-consanguineous parents. He inherited the V84M mutation in *KCNJ10* from his mother. The neurodevelopment was typical up to 12 months of age, when a regression became evident and, 6 months later, the child received a diagnosis of ASD. At 6 years, he experienced complex partial seizures that partially remitted under valproate. EEGs showed bilateral frontal paroxysms while awake and asleep, while brain MRI scan was normal.

Supplementary Table S1

Gain-of-function *KCNJ10* variants (R18Q, R348H, V84M) vs WT

	**WT** (n=166)	***KCNJ10*** **Variants** (n=9)	**Test**	**Effect size**	**p**
**Age**					
mean (SD)	7.5 (4.2)	7.7 (2.4)	t=-0.172	-	0.863
**Gender**					
Male	143 (86.1%)	8 (88.9%)	$\chi^{2=}$0.054	Φc=0.018	0.816
Female	23 (13.9%)	1 (11.1%)			
**ASD diagnosis**					
Autism	51 (30.7%	1 (11.1%)	$\chi^{2=}$1.839	Φc=0.103	0.399
PDDNOS	112 (67.5%)	8 (88.9%)			
Asperger	3 (1.8%)	-			
**Language Development**					
Normal	32 (19.3%)	1 (11.1%)	$\chi^{2=}$2.573	Φc=0.121	0.276
Delayed	84 (50.6%)	7 (77.8%)			
Absent	50 (30.1%)	1 (11.1%)			
**Cognitive Development**					
Normal-Borderline	71 (44.9%)	1 (11.1%)	$\chi^{2=}$3.975	Φc=0.154	0.137
Mild-Moderate Delay	55 (34.8%)	5 (55.6%)			
Severe Delay	32 (20.3%)	3 (33.3%)			
**Self/hetero injurious behavior**					
Yes	91 (55.8%)	5 (55.6%)	$\chi^{2=}$0.000	Φc=0.001	0.987
No	72 (44.2%)	4 (44.4%)			
**Frustration intolerance**					
Yes	118 (72.4%)	8 (88.9%)	$\chi^{2=}$1.185	Φc=0.083	0.276
No	45 (27.6%)	1 (11.1%)			
**Sleep disorders**					
Yes	44 (27.3%)	2 (22.2%)	$\chi^{2=}$0.113	Φc=0.026	0.737
No	117 (72.7%)	7 (77.8%)			
**Macrocephaly**					
Yes	50 (30.7%)	-	$\chi^{2=}$3.042	Φc=0.134	0.081
No	113 (69.3%)	7 (100%)			
**Family History of Epilepsy**					
Yes	48 (29.6%)	2 (22.2%)	$\chi^{2=}$0.226	Φc=0.036	0.634
No	114 (70.4%)	7 (77.8%)			
**Family History of ASD**					
Yes	28 (17.2%)	2 (22.2%)	$\chi^{2=}$0.151	Φc=0.030	0.698
No	135 (82.8%)	7 (77.8%)			

WT= Wild type; t=t-test; χ^2=the Pearson chi-squared test; Φc= Cramers' phi coefficient

Supplementary Table S2

Gain-of-function *KCNJ10* variants (R18Q, R348H, V84M) vs WT. Children harbouring the neutral p.R271C were removed from the WT group

	WT (n=156)	*KCNJ10* Variants (n=9)	Test	Effect size	p
**Age**					
mean (SD)	7.4 (4.2)	7.7 (2.4)	t=-0.241	-	0.810
**Gender**					
Male	133 (85.3%)	8 (88.9%)	χ^{2}=0.090	Φc=0.023	0.764
Female	23 (14.7%)	1 (11.1%)			
**ASD diagnosis**					
Autism	50 (32%)	1 (11.1%)	χ^{2}=1.942	Φc=0.108	0.379
PDDNOS	104 (66.7%)	8 (88.9%)			
Asperger	2 (1.3%)	-			
**Language Development**					
Normal	29 (18.6%)	1 (11.1%)	χ^{2}=2.337	Φc=0.119	0.311
Delayed	81 (51.9%)	7 (77.8%)			
Absent	46 (29.5%)	1 (11.1%)			
**Cognitive Development**					
Normal-Borderline	68 (45.9%)	1 (11.1%)	χ^{2}=4.187	Φc=0.163	0.123
Mild-Moderate Delay	51 (34.5%)	5 (55.6%)			
Severe Delay	29 (19.6%)	3 (33.3%)			
**Self/hetero injurious behavior**					
Yes	87 (56.9%)	5 (55.6%)	χ^{2}=0.006	Φc=0.006	0.939
No	66 (43.1%)	4 (44.4%)			
**Frustration intolerance**					
Yes	112 (73.2%)	8 (88.9%)	χ^{2}=1.089	Φc=0.082	0.297
No	41 (26.8%)	1 (11.1%)			
**Sleep disorders**					
Yes	43 (28.5%)	2 (22.2%)	χ^{2}=0.164	Φc=0.032	0.685
No	108 (71.5%)	7 (77.8%)			
**Macrocephaly**					
Yes	47 (30.7%)	-	χ^{2}=3.045	Φc=0.138	0.081
No	106 (69.3%)	7 (100%)			
**Family History of Epilepsy**					
Yes	45 (29.4%)	2 (22.2%)	χ^{2}=0.213	Φc=0.036	0.644
No	108 (70.6%)	7 (77.8%)			
**Family History of ASD**					
Yes	28 (18.3%)	2 (22.2%)	χ^{2}=0.087	Φc=0.023	0.769
No	125 (81.7%)	7 (77.8%)			

WT= Wild type; t=t-test; χ^2=the Pearson chi-squared test; Φc= Cramers' phi coefficient

Supplementary Table S3

Gain-of-function *KCNJ10* variants (R18Q, R348H, V84M) vs WT. Children harbouring the neutral p.R271C were removed from the WT group

	WT (n=156)	*KCNJ10* Variants (n=9)	Test (χ^2)	Effect size (Φc)	p
**History of Seizures**					
Yes	51 (32.7%)	5 (55.6%)	1.984	0.110	0.159
No	105 (67.3%)	4 (44.4%)			
**Type of Seizures**					
Focal	32 (62.7%)	1 (20%)	14.035	0.501	**0.001***
Generalized	16 (31.4%)	1 (20%)			
Spasms	3 (5.9%)	3 (60%)			
**EEG abnormalities**					
Yes	113 (76.9%)	7 (77.8%)	0.490	0.056	0.484
No	34 (23.1%)	2 (22.2%)			
**Site of EEG abnormalities**					
Anterior	39 (34.5%)	3 (42.9%)	2.800	0.153	0.424
Posterior	17 (15%)	0			
Temporal	15 (13.3%)	0			
Multifocal/Diffuse	42 (37.2%)	4 (57.1%)			
**Type of EEG abnormalities**					
Paroxysms	57 (50.5%)	4 (57.1%)	1.242	0.102	0.537
Focal slowing	17 (15%)	0			
Both	39 (34.5%)	3 (42.9%)			
**Stereotyped Behaviours**					
Yes	137 (89%)	3 (37.5%)	4.921	0.174	**0.027****
No	17 (11%)	5 (62.5%)			
**Regulation Disorder of Sensory Processing**					
Yes	105 (68.6%)	9 (100%)	4.012	0.157	**0.045*****
No	48 (31.4%)	0			

WT= Wild type; χ^2=the Pearson chi-squared test; Φc= Cramers' phi coefficient; **KCNJ10* variants were significantly associated with epileptic spasms; ** *KCNJ10* variants showed stereotyped behaviours less frequently than WT; *** *KCNJ10* variants showed a Regulation Disorder of Sensory Processing more frequently than WT

Supplementary Fig. S1

WT and mutated Kir4.1 expression and distribution in U251 astrocytoma cells. **(a)** Immunofluorescence stainings of U251 astrocytoma cells with anti-Kir4.1 pAb (red) and FITC-conjugated phallacidin (green) to stain actin filaments show that the endogenous Kir4.1 channels are mainly distributed in cytoplasmic perinuclear area and scarcely at plasma membrane. Scale bars: 10 µm. **(b)** RT-PCR analysis using primers to detect specifically recombinant Kir4.1 mRNA expression (Forward: Xpress epitope/pcDNA 3.1/His, Life technologies; Kir4.1 Reverse: TCAGACATTGCTGATGCGCAC) in stably infected U251 cells reveals no differences between WT (1) and R18Q Kir4.1 (2) expressing cells. GAPDH housekeeping gene normalizes the amount of template used

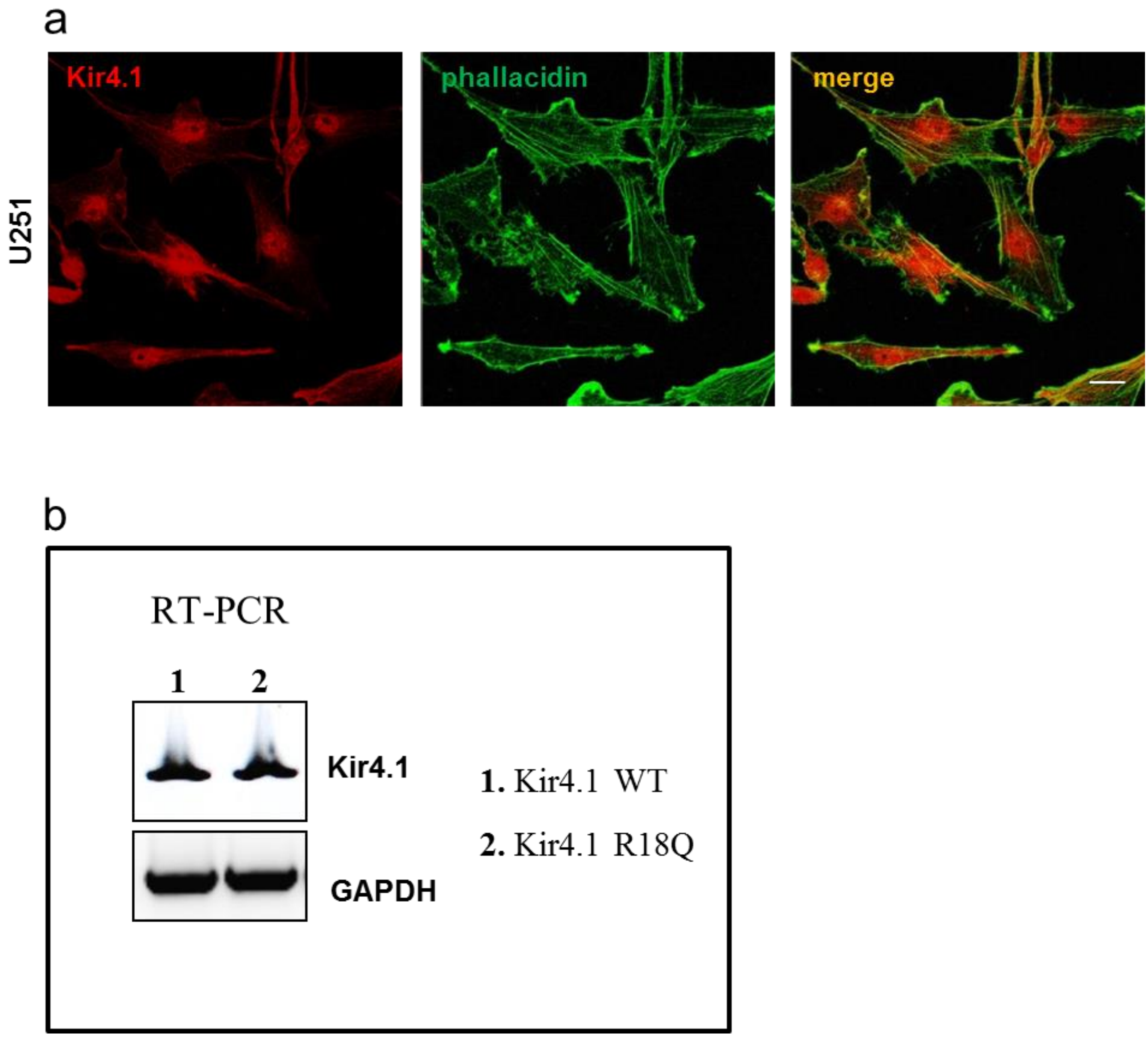

Supplementary Fig. S2

Double immunofluorescence stainings with anti-Kir4.1 pAb (red) and anti-synt mAb (green) in U251 cells expressing WT (upper panels) or R18Q (lower panels) Kir4.1 reveal a partial colocalization of Kir4.1 and syntrophin in the plasma membrane and in the cytoplasm of both astrocytoma cell lines. Compared to Kir4.1 WT expressing cells, a larger number of R18Q^{+} cells shows colocalization of syntrophin and Kir4.1 in both cytoplasm and plasma membrane (arrowheads). Scale bars: 10 µm

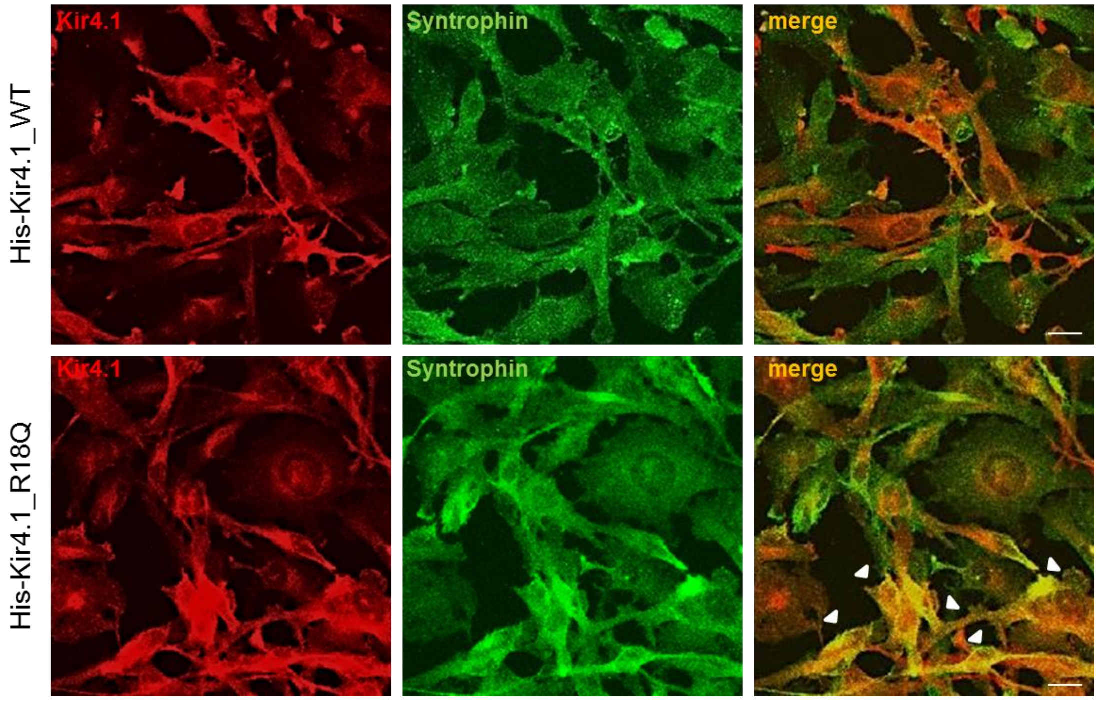

Supplementary Fig. S3

Characterization of astrocytoma cells expressing WT, R271C and R348H Kir4.1 channels. **(a)** WB analysis of cytosolic (CYT) and membrane (MEM) protein fractions derived from astrocytoma cells stably expressing WT and Kir4.1 mutations R271C and R348H. No significant differences in Kir4.1 expression levels were observed between WT and mutated Kir4.1 expressing cells. Actin is used as internal loading control. Molecular weight markers are on the left (kDa). **(b)** Co-immunofluorescences of astrocytoma cells expressing WT or mutated channels with anti-Xpress mAb (red) and FITC-conjugated phallacidin (green) show no differences in the distribution of WT, R271C and R348H channels in the cytoplasm and plasma membrane of U251 cells. Scale bar: 10 µm

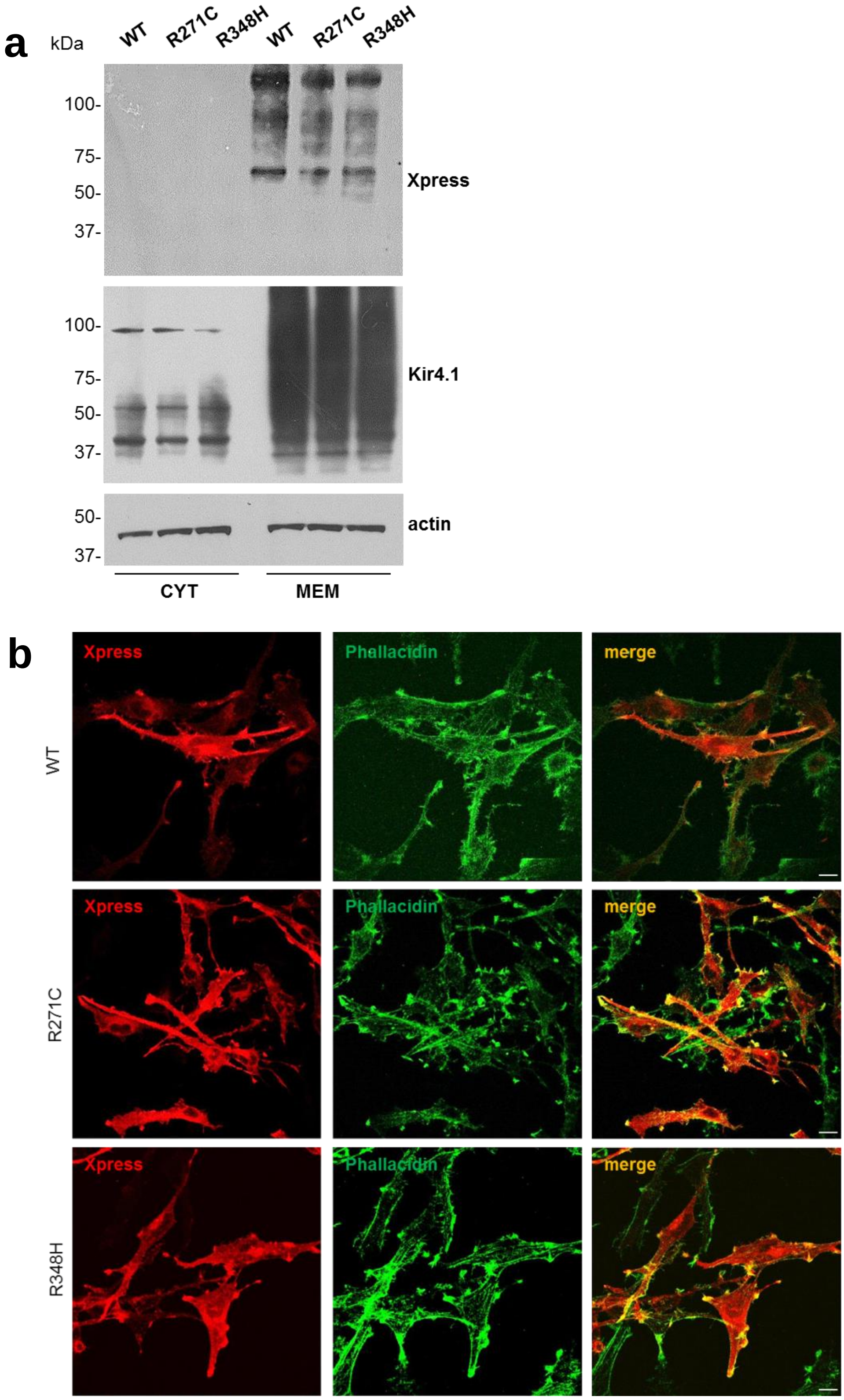

Supplementary Fig. S4

Co-purification of Kir4.1 channel's interactors. WB analysis of Kir4.1 channel interactors identified by Histidine (His) co-purification of astrocytoma cells expressing His-tagged WT or mutated channels. Eluates derived from His pull-down, performed with astrocytoma cells infected with the empty vector, are used as a control for non specific binding to NiNTA-resin (U251). Input lanes represent the starting protein extracts before His pull-down. Interactors have been eluted from NiNTA-resin using imidazole (200 mM). WT and mutated channels co-purify similarly with Kir2.1 and Kir5.1. In this experiment no interaction is observed with connexin-43 and aquaporin-4 (AQP4). One representative experiment out of four is shown. Molecular weight markers are indicated on the left (kDa)

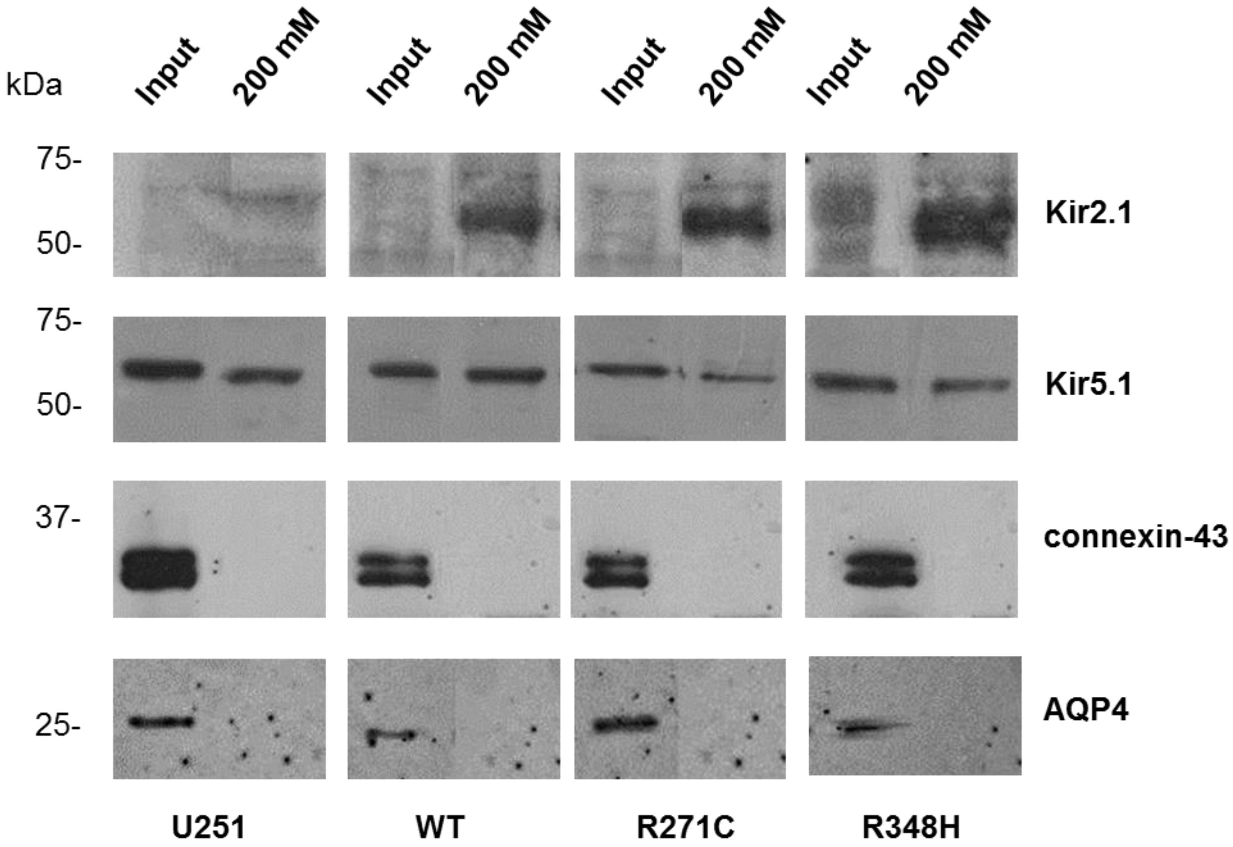

Supplementary Methods

Homology modeling. The 3D structure of Kir4.1 was built as previously described[1]. The X-ray structure of the G Protein-Gated Inward Rectifier GIRK1 (PDB id.: 1N9P) was used as a template[2]. Only residues 23-48 and 176–361 of the Kir4.1 primary structure could be aligned with the corresponding segments of the X-ray template. Twenty homology models were generated and scored against the minimum number of constraint violations. Among them, the five with lowest energy models were selected and analyzed using Procheck[3]. The final model was chosen according to the highest percentage of residues in the allowed region of the Ramachandran plot (>90%). The R348H mutant was generated by substituting the Arginine-348 side-chain with that of Histidine using VMD[4]. The final model was further minimized to reduce steric hindrance with neighboring atoms using GROMACS4 and the GROMOS96 force field[5].

1. Sicca, F. *et al.* Autism with seizures and intellectual disability: Possible causative role of gain-of-function of the inwardly rectifying K+ channel Kir4.1. *Neurobiol. Dis.* **43,** 239-247 (2011).
2. Nishida M, MacKinnon R (2002) Structural basis of inward rectification: cytoplasmic pore of the G protein-gated inward rectifier GIRK1 at 1.8 A resolution. Cell 111:957-965. doi: 10.1016/S0092-8674(02)01227-8
3. Laskowski RA, MacArthur MW, Moss DS, Thornton JM (1993) PROCHECK: a program to check the stereochemical quality of protein structures. J App Cryst 26:283-291. doi: 10.1107/S0021889892009944
4. Humphrey W, Dalke A, Schulten K (1996) VMD: visual molecular dynamics. J Mol Graphics 14:33-38. doi: 10.1016/0263-7855(96)00018-5
5. Hess B, Kutzner C, van der Spoel D, Lindahl E (2008) GROMACS 4: algorithms for highly efficient, load-balanced, and scalable molecular simulation. J Chem Theor Comp 4:435-447. doi: 10.1021/ct700301q

Supplementary Fig. S5

3D-homology model of a Kir4.1 channel indicating the position of the R348H mutation. **(a)** Crystal structure of the cytoplasmic domain of the channel showing the close interactions taking place between the N- and C-terminal regions. The position of the Arg-18 residue in the N-terminal region could not be determined due to lack of structural data. The first residue available in the crystal structure is Arg-27. **(b)** View of the channel indicating the position of the Arg-348 and the Arg348His mutation shown as sticks in the heterozygous state

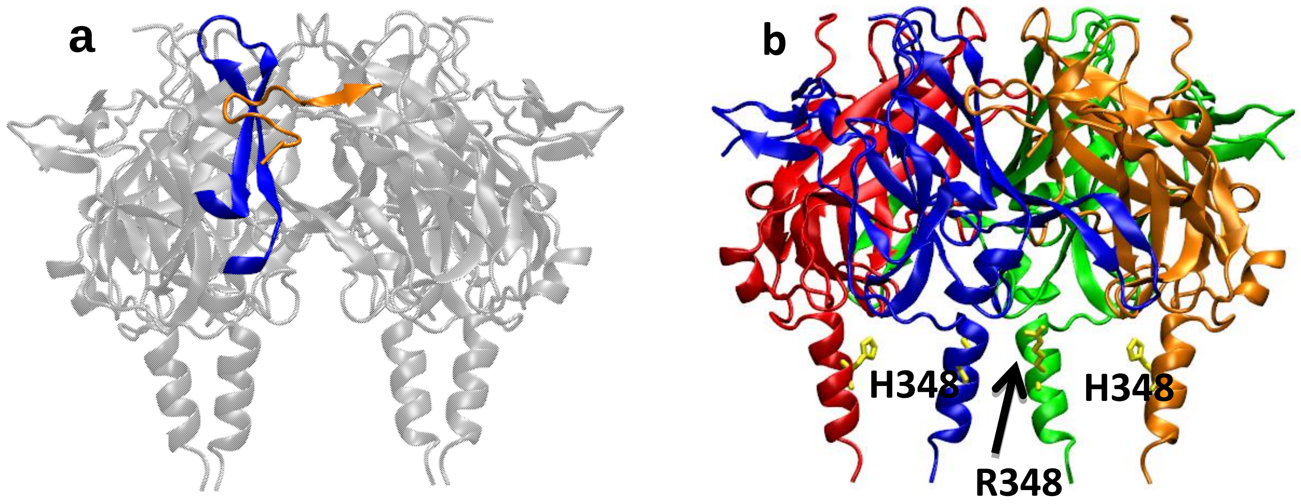

Supplementary Fig. S6

Ribbon representation of the 3D homology structure of the Kir4.1 channel (front-view) **(a)** based on available crystal structure data (see Methods), showing the location of the R271 and the R271C variant (hard-spheres) in the cytoplasmic domain of the Kir4.1 channel (each subunit is coloured differently). Dashed lines delimit the transmembrane spanning region. **(b)** Bottom-up view of the intracellular domain for the heterozygous Kir4.1 channel, showing two WT (R271) and two C271 subunits

.

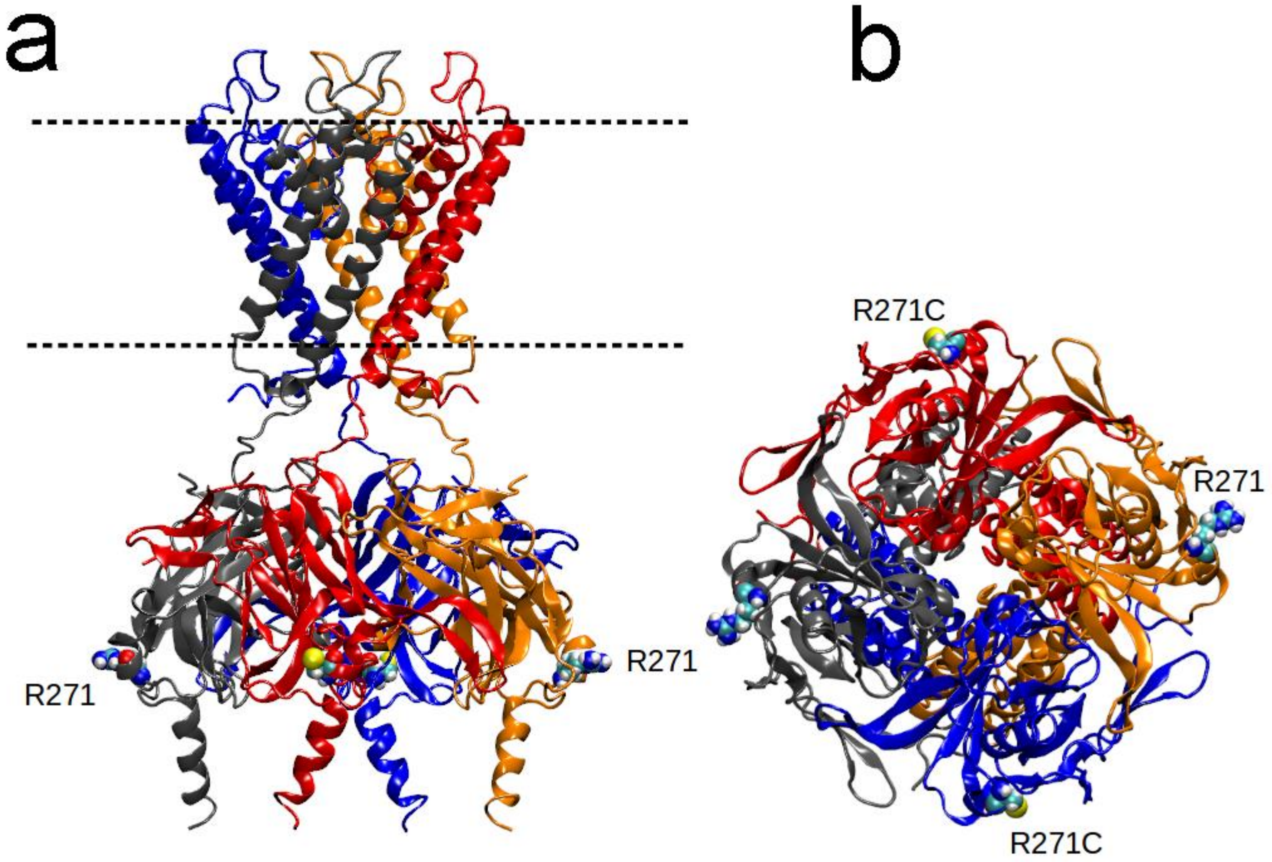

Supplementary Fig. S7

In vivo modelling of Kir4.1 mutations in zebrafish. The graph shows the percent of flicks at 30 hpf (registration time 30sec), in uninjected embryos, and in embryos injected with either the WT, or R18Q, or WT+R18Q human mRNA. Embryos injected with R18Q, and with equimolar amounts of R18Q+WT human mRNA showed an increased rate of spontaneous contractions, compared to uninjected and to WT alone mRNA injected embryos, suggesting a possible "toxic" GoF effect. ** $p<0.05$; ** $p<0.01$

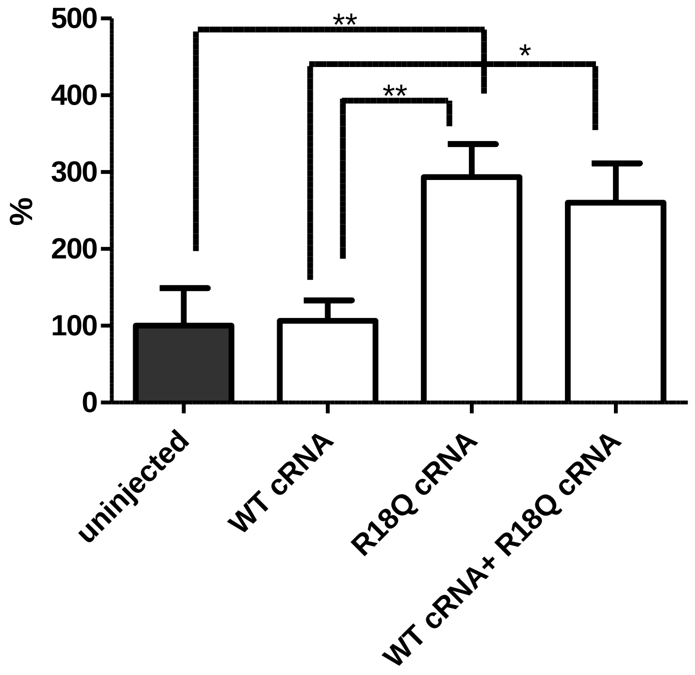

Supplement: Supplementary Information [file srep34325-s1.pdf]
